# xProtCAS: A Toolkit for Extracting Conserved Accessible Surfaces from Protein Structures

**DOI:** 10.3390/biom13060906

**Published:** 2023-05-30

**Authors:** Hazem M. Kotb, Norman E. Davey

**Affiliations:** Division of Cancer Biology, The Institute of Cancer Research, 237 Fulham Road, London SW3 6JB, UK

**Keywords:** protein, evolution, structure, interactions, conserved surface discovery, graph theory

## Abstract

The identification of protein surfaces required for interaction with other biomolecules broadens our understanding of protein function, their regulation by post-translational modification, and the deleterious effect of disease mutations. Protein interaction interfaces are often identifiable as patches of conserved residues on a protein’s surface. However, finding conserved accessible surfaces on folded regions requires an understanding of the protein structure to discriminate between functional and structural constraints on residue conservation. With the emergence of deep learning methods for protein structure prediction, high-quality structural models are now available for any protein. In this study, we introduce tools to identify conserved surfaces on AlphaFold2 structural models. We define autonomous structural modules from the structural models and convert these modules to a graph encoding residue topology, accessibility, and conservation. Conserved surfaces are then extracted using a novel eigenvector centrality-based approach. We apply the tool to the human proteome identifying hundreds of uncharacterised yet highly conserved surfaces, many of which contain clinically significant mutations. The xProtCAS tool is available as open-source Python software and an interactive web server.

## 1. Introduction

The characterisation of the human interactome has been fundamental for our understanding of cellular processes. Tens of thousands of human protein-protein interactions (PPIs) have been detected using a range of experimental techniques [1,2]. To date, most PPI data describe the binary interaction between two full-length proteins. Recent experimental and computational approaches have defined stable complexes, thereby building higher-order PPI networks more closely matching the protein organisation in the cell [3,4]. The interfaces of these interactions have been characterised at the amino acid resolution for only a minority of PPIs. The molecular detail of a PPI interface is extremely valuable for a detailed characterisation of protein function or, in some cases, as potential therapeutic targets. Consequently, there is a strong need for an amino acid resolution interactome. Numerous experimental approaches have been applied to characterise PPI interfaces at this level of detail, including structural, mutagenesis, or biophysical assays. However, they are generally expensive, time-consuming, and low throughput. As a result, interface identification is often supported by computational approaches to pinpoint residues or regions likely to drive PPIs [5].

Various sequence-based and structure-based features have been analysed to discover protein interfaces [6,7,8,9]. Sequence conservation tends to be the strongest discriminator of residue functionality [10,11]. A common observation is that protein interfaces are often under functional constraints and less likely to accumulate mutations. Consequently, as surface residues often lack the strong structural constraints of the hydrophobic core residues supporting a protein fold, functionally constrained interaction surfaces can often be observed as accessible surfaces with relatively high conservation compared to the remaining protein surface [12]. Many computational studies have focused on the discovery of conserved accessible surfaces as these regions strongly overlap real interaction interfaces [13,14]. The residues that form interfaces in folded domains are often non-sequential with atoms contributed from amino acids scattered through the primary sequence of the protein. Consequently, these algorithms generally include structural information and search for clusters of conserved residues proximal in three-dimensional space.

Several conservation-based interface prediction tools have been developed, and despite the similar underlying goal, each approach differs on the algorithm level. PatchFinder [15,16] searches for functional regions by identifying the largest and most conserved cluster of residues using a heuristic greedy search algorithm not guaranteed to converge to the optimal solution. FuncPatch [17,18] uses a Gaussian process as a sampling proxy for the 3D structure to encode spatial correlation between conservations in the three-dimensional space relying on the smoothness property of the Gaussian priors. It uses maximum likelihood to estimate the underlying Gaussian parameters given a multiple sequence alignment and phylogenetic tree. FuncPatch smoothes scores based on the neighbourhood but does not value the cluster size nor guarantees that residues in a more conserved cluster have larger scores. ConSurf [19,20] uses Bayesian estimation of conservation scores for each position in a multiple sequence alignment given a phylogenetic tree and projects these residue scores onto a three-dimensional structure to enable the user to identify a conserved accessible surface. Therefore, ConSurf does not use the three-dimensional structure to calculate the residue scores.

An alternative approach to conservation-based methods is to study binding-site-level features based on geometric properties and identify binding sites by finding protein surface cavities [21]. Other approaches include energetic methods, template-based methods, or a combination of methods [22,23,24,25]. Studies increasingly use machine learning or deep learning to build trained models [24,26,27]. Evolutionary information is also used in these approaches; for example, template-based methods depend on sequence homology and structure alignments [28]. Furthermore, some studies integrate evolutionary information with geometric properties [29], while others use evolutionary information to filter pockets discovered geometrically [30]. Generally, when conservation is combined with other approaches, the discriminatory power of the approach is improved [31]. Finally, when both binding partners in the interface are known, a range of docking-based approaches can be applied, most notably the recently released AlphaFold-Multimer [32].

Intuitive descriptive tools for defining conserved surfaces on protein structures simplify the task of biologists by characterising protein functionality. The advent of deep learning approaches for protein structure prediction has resulted in high-quality tertiary structural models for any protein. In this study, we have developed a novel method, xProtCAS (e**x**tracting **Prot**ein **C**onserved **A**ccessible **S**urfaces), to take structurally resolved or modelled protein regions and define conserved accessible surfaces by combining the structural and evolutionary data. The xProtCAS framework is available as open-source software and a user-friendly web server. The tool has been applied to produce a dataset of putative functional surfaces in the human proteome.

## 2. Methods

The xProtCAS framework is a pipeline for the discovery of clusters of conserved residues on the surface of folded structural modules as a proxy for functional interfaces. The xProtCAS pipeline integrates information on residue solvent accessibility and conservation with topological data on residue proximity in three-dimensional space using graph-based methods to determine proximal clusters of relatively conserved residues on a protein’s surface. The key use case of the xProtCAS framework is the analysis of human AlphaFold2 [33,34] models taking a UniProt identifier as input. However, the standalone version can use either AlphaFold2 models or PDB structures. The output is a set of conservation and accessibility metrics for the protein and an annotated and scored conserved surface on each autonomous structural module of the protein.

### 2.1. Framework for the Discovery of Conserved Protein Surface

**Figure 1:**
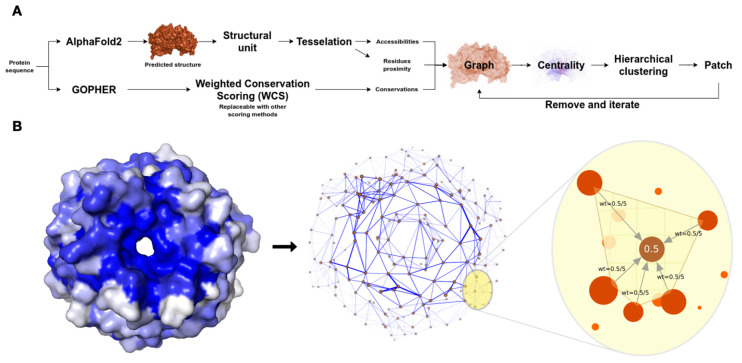
Overview of the xProtCAS framework. (**A**) Schema of the steps in the xProtCAS pipeline. (**B**) Schema representing the encoding of a structural module as a graph in the xProtCAS framework. The graph encodes the proximity between residues, accessibility, and conservation of the structural module. Nodes are accessible residues and adjacent residues are connected by edges. The blue colour of the surface structure representation of the structural module reflects the conservation of residues. The conservation scores are encoded on the graph as edge weights demonstrated by edge thickness in the graph panel. Residues sharing a tetrahedron in the three-dimensional Delaunay triangulation are considered neighbours. Each residue has incoming edges from neighbouring residues. The weight of the edge depends on the conservation of the residue and the number of neighbours. For example, in a residue with a conservation score of 0.5 and five adjacent neighbours, each incoming edge weights 0.5/5. Each accessible residue is in one tetrahedron at least, resulting in the whole structural module being connected in a single graph.

As shown in Figure 1A, the workflow of the xProtCAS framework includes eight major steps: (i) definition of the autonomous structural modules of a protein; (ii) calculation of the residue-centric accessibility and topology metrics for the structural module; (iii) calculation of the per residue conservation scores; (iv) creation of an edge-weighted directed graph encoding the structural and evolutionary properties for the structural module; (v) calculation of eigenvector centrality scores; (vi) definition of the conserved accessible surfaces using hierarchical clustering; and (vii) scoring and (viii) annotation of the conserved accessible surfaces.

#### 2.1.1. Definition of the Autonomous Structural Modules of the Structural Model

In the first step of the pipeline, the AlphaFold2 model of a full-length protein of interest is retrieved from the AlphaFold protein structure database [33,34] (https://alphafold.ebi.ac.uk/). The structural model is preprocessed to define autonomous structural modules. This allows intramolecular interface surfaces to be ignored and each autonomous functional unit to be analysed separately. Autonomous structural modules are extracted from the AlphaFold2 structure model by running a graph-based community detection algorithm on the AlphaFold2 predicted aligned error (PAE) matrix [35]. First, a graph is built from residues with AlphaFold2 per-residue confidence (pLDDT) > 70, where residues are nodes and edges are placed between residues with AlphaFold2 PAE less than 5 Å. The edges are weighted based on the inverse of the predicted aligned error, and a greedy modularity maximisation algorithm [36] is used to detect communities. Modularity measures the quality of communities in a graph by calculating the difference between two components. The first represents the edges that fall within the detected communities, and the second counts for the expectation of those edges happening randomly (the null model). The best communities are those maximising that difference. The resolution, the weight of the null model in the modularity equation, controls the size of the detected communities. A resolution of zero gives no weight to the null model and counts only the intra-community edges, resulting in one community containing the whole protein in the graph [36]. Higher resolution values favour smaller communities. The resolution of the modularity maximisation algorithm was optimised on a dataset of 832 human E3 ligases manually collected from the literature (Table S1). By iteratively decreasing the resolution from 1 to 0 by 0.01 steps, we defined an optimised resolution that minimised the size of structural modules but ensured that Pfam domains [37] were not split between different structural modules (Figure S1). Finally, small communities with less than 30 residues, the lower bound for a stably folded domain, were excluded. The structural modules derived from this step are processed independently in the remainder of the analysis pipeline.

#### 2.1.2. Defining the Residue Accessibility and Topology of a Structural Module

The residue accessibility of each structural module is calculated by Delaunay triangulation which generates a triangle-based tessellation of a protein surface. This approach has the advantage, relative to classical solvent accessible surface area (SASA) metrics, of discriminating between side chain and backbone accessibility. Delaunay triangulation [38,39] takes the three-dimensional coordinates of each heavy atom in the structural module and produces a set of non-overlapping tetrahedra (triangular pyramids composed of four triangular faces). The centre of each heavy atom is considered a vertex and is present in at least one tetrahedron in the convex hull (the smallest set of vertices enclosing the whole structural module). Atoms on the surface of the structural module can be extracted by finding vertices in faces present in exactly one tetrahedron. The xProtCAS pipeline ignores backbone accessibility and considers an amino acid accessible if at least one of its side chain heavy atoms is accessible (Table S2). The 3D Delaunay triangulation calculation is based on the implementation by Nimrod et al. [16]. Triangulation is also used to identify a residue’s neighbours as those residues with surface atoms in a shared tetrahedron.

#### 2.1.3. Calculating per Residue Conservation Scores of a Structural Module

Conservation scores are derived from orthologue alignments created using the GOPHER orthologue discovery software [40] on a database of model organism sequences from the UniProt resource [41]. The orthologous sequences were aligned using the ClustalO multiple sequence alignment software [42], and guide trees were generated using the tree-building function of ClustalW [43]. The xProtCAS pipeline calculates a classical column-based weighted conservation score (WCS) as defined in SLiMPrints [44]. The contribution of each residue at a given position in the alignment is weighted by the Clustal guide tree in the WCS score to increase the contribution of conserved residues in more divergent orthologues.

#### 2.1.4. Constructing an Edge-Weighted Directed Graph Representation of a Structural Module

A directed graph is constructed encoding the residue accessibility, residue conservation, and residue proximity in the three-dimensional space of the structural module (Figure 1B). The topology, accessibility, conservation (TAC) graph contains only residues with accessible side chain heavy atoms. Directed edges are added between adjacent residues where adjacency is defined based on surface atoms that share the same tetrahedron in the Delaunay triangulation graph produced during accessibility calculation [16]. Each incoming edge is weighted by the node’s conservation score, divided by the number of incoming edges to normalise the number of proximal residues (Figure 1B).

#### 2.1.5. Calculating Eigenvector Centrality Scores of a Structural Module

Although filtering residues using a simple conservation score cutoff might seem sufficient for finding functional regions, it yields several residues spread on the surface of the protein. Hence, finding conserved patches instead of single residues is crucial to pinpoint the more likely functional regions. Eigenvector centrality [45] measures a node’s importance, in this case, encoded as residue conservation, but also integrates the importance of surrounding nodes. Therefore, a node connected to more highly conserved nodes receives a higher centrality score [46]. Eigenvector centrality scores are calculated for each residue in the TAC graph. The definition of a node’s transitive influence in the TAC graph allows the discrimination of groups of proximal conserved residues to identify conserved accessible surfaces on a structural module.

#### 2.1.6. Defining Conserved Accessible Surfaces of a Structural Module

Eigenvector centrality scores are processed using a hierarchical clustering approach to extract the most conserved cluster of residues on the protein surface. Initially, each node in the graph is considered a separate cluster on its own. Next, pairs of clusters are successively combined into larger clusters by minimising the variance of centrality scores in the merged clusters. The hierarchical clustering produces two low variance clusters, one with the highest central scoring residue nodes and the other with the remaining surface accessible residue nodes in the TAC graph. Finally, the connected residues are extracted from the highest centrality-scoring cluster by starting with the node with the highest centrality score and recursively finding connected neighbours in the graph that are also in the high-scoring cluster to define the most conserved accessible surface.

#### 2.1.7. Evaluation Metrics of the Extracted Patch

Three distinct conservation metrics are calculated evaluating the conserved accessible surface: (i) absolute patch conservation: the average residue WCS conservation of the patch representing the absolute conservation of the patch in the orthologous species set; (ii) relative patch conservation: the difference between the average of WCS conservation in the patch and the average of WCS conservation in the non-patch surface representing the relative conservation of the pocket compared to the remainder of the structural module surface; and (iii) the associated *p*-value of the relative patch conservation *p*-value calculated as a Mann–Whitney U test for the alternative hypothesis that the distribution underlying patch conservations is stochastically greater than the distribution underlying non-patch conservations.

#### 2.1.8. Annotations of the Pockets with Functional Data

Identified conserved accessible surfaces are cross-referenced with functional annotation from a range of sources. Domain family overlap information is defined using Pfam domains [37] data collected from UniProt [41]. The intersection between defined conserved accessible surfaces with residues in experimentally characterised interfaces is mapped based on extraction of protein interfaces from the PDB structures where interface residues were determined as residues with heavy atoms within less than 6 Å distance from the bound partner. Overlapping active sites are collected from the UniProt resource [41]. Disease-relevant mutations data in the predicted pockets are collected from the EBI Protein API [47] and UniProt [41] and classified based on clinical significance annotation (Pathogenic, Likely Pathogenic, Disease, Risk factor, Association, Protective, Drug response, Affects) as in PepTools [48]. Post-translational modifications overlapping the defined conserved accessible surfaces are collected from Phospho.ELM [49], PhosphoSitePlus [50], Ochoa et al. [51], and UniProt [41].

### 2.2. Defining Multiple Pockets per Structural Module

As proteins or domains can have multiple interaction interfaces, centrality scoring, and hierarchical clustering can be performed iteratively after the removal of the residues in the initial patch from the graph representation to define additional interfaces.

### 2.3. Processing PDB Structures

The xProtCAS pipeline can be applied beyond AlphaFold models to any PDB structures. All pipeline steps except for the “definition of the autonomous structural modules” are performed for PDB structure analyses. When required, PDB structures are mapped to UniProt [41] sequence position-centric conservation scores using SIFTS [52] and the PDBe REST API [53].

### 2.4. PDB Benchmarking Dataset

The xProtCAS pipeline was optimised and benchmarked on three datasets of PDB structures (Table S3): (i) 407 domain-domain interaction interfaces from the MaSIF-site dataset [54], (ii) 522 SLiM-domain interaction interface dataset extracted from PDB and filtered for redundancy (see Supplementary material note 1), and (iii) 100 active sites in structures from the two previous datasets. The MaSIF-site dataset was filtered to remove structures with >30% identity to structures in the unfiltered SLiM-domain interaction interface dataset. Interface residues, defined as *pocket residues*, were determined in each set as residues with heavy atoms less than 6 Å distant from the bound partner. The remainder of the residues on the protein surface not found in the *pocket* residues set are defined as *non-pocket* residues. Residues that are defined by the Delaunay triangulation as accessible are defined as *surface* residues. Residues that are filtered by the Delaunay triangulation as inaccessible with side chains in the hydrophobic core of the protein are defined as *core* residues.

### 2.5. Human Proteome Analysis

The xProtCAS tool was applied to 20,395 proteins of the Human Proteome (UniProt reviewed human proteins with no fragments, release 2021_02) [41] to define structural modules and identify conserved accessible surfaces.

### 2.6. Availability

All the pipeline steps were implemented in Python. A stand-alone open-source software with the core functionality of the pipeline is available at https://github.com/hkotb/xprotcas. The xProtCAS framework is also accessible as a web server at http://slim.icr.ac.uk/projects/xprotcas.

## 3. Results

### 3.1. Evaluating Residue Conservation for Identifying Binding Surfaces

The PDB benchmarking dataset represents an evaluation set to benchmark xProtCAS’s ability to define conserved accessible surfaces and whether these regions are likely to represent functional sites on proteins.

#### 3.1.1. Weighted Residue-Based Conservation Scoring (WCS) Benchmarking

**Figure 2:**
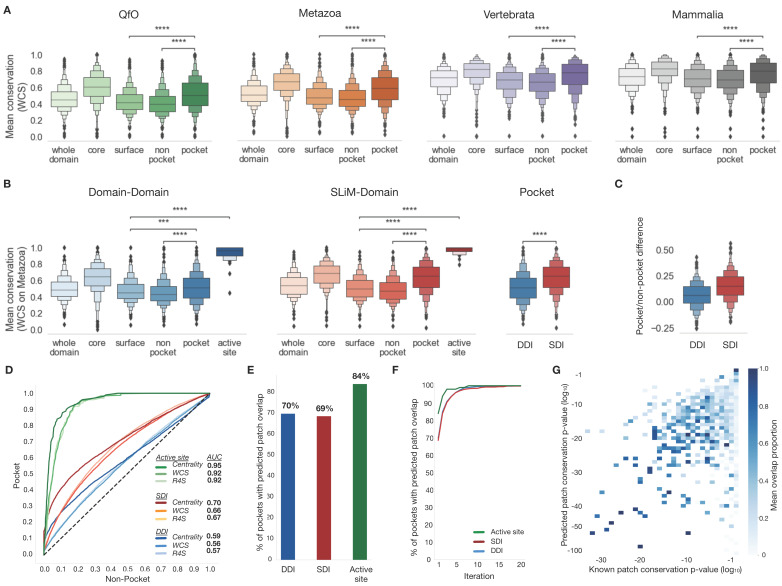
The evaluation of per residue conservation and per patch centrality scores of interaction interfaces and active sites. (**A**) A boxen plot of the mean Weighted Conservation Scores (WCS) conservation calculated for the whole domain (core + surface), the inaccessible core of the domain, the surface of the domain (pocket + non-pocket), the pocket, and the non-pocket regions of the domain. The conservation scores are evaluated on orthologue alignments from four orthologue sets with different levels of divergence. Asterisks show the level of significance measured in *p*-values of the Mann-Whitney-Wilcoxon test two-sided with Bonferroni correction (***: 10^−4^ < *p*-value ≤ 10^−3^, ****: *p*-value ≤ 10^−4^). (**B**) Boxen plots comparing the mean WCS conservation of pockets from the rest of the surface on the different interface types. (**C**) Boxen plots comparing the mean WCS conservation between the pocket and non-pocket regions. (**D**) ROC curves comparing two conservation scores (Rate4Site (R4S) and WCS conservation) and the eigenvector centrality scores for their ability to discriminate between interface or active sites residues and other residues on the surface of SDI and DDI structures. (**E**) Evaluation of the proportion of xProtCAS conserved accessible surfaces overlapping validated functional surfaces. (**F**) Evaluation of the cumulative proportion of xProtCAS conserved accessible surfaces overlapping validated functional surfaces over multiple iterations. (**G**) Heatmap of mean overlap proportion of xProtCAS conserved accessible surfaces with validated functional surfaces as a function of the significance level of the conservation represented in *p*-values of the known interfaces and the predicted patches.

We evaluated weighted residue-based conservation scoring (WCS) on orthologue alignments from four sets of proteins from proteomes of species with different levels of evolutionary divergence (Mammalia, Vertebrata, Metazoa, Quest for Orthologs (QfO) [55], see supplementary material for a list of species in each database). The weighted residue-based conservation scoring can discriminate between the pocket and non-pocket residues in all orthologue alignment sets (Figure 2A). The metazoa-based alignment shows the most discriminatory power between the pocket and non-pocket regions (*p*-value: 5.66 × 10^−44^) compared to the three other orthologue alignments sets (QfO *p*-value: 1.95 × 10^−40^, Vertebrata *p*-value: 1.31 × 10^−36^, Mammalia *p*-value: 1.45 × 10^−25^). Next, we evaluated metazoa-based scoring separately on the domain-domain (DDI), SLiM-domain (SDI), and the active site PDB benchmarking dataset. We observed a significant difference between the pocket and non-pocket residues for both DDI and SDI sets (DDI *p*-value: 3.44 × 10^−8^, SDI *p*-value: 5.27 × 10^−44^) (Figure 2B,C). Interestingly, SDI pockets are clearly more conserved than DDI interfaces in the benchmarking dataset (*p*-value: 1.94 × 10^−14^) (Figure 2B,C). Active sites are the most conserved set in all cases and are significantly more conserved than surface residues in both interface sets (DDI *p*-value: 5.53 × 10^−26^, SDI *p*-value: 1.21 × 10^−33^) (Figure 2B). Finally, we compared the WCS-weighted residue-based conservation scoring to the Rate4Site scores [56,57] from ConSurf [19,20]. The WCS scoring scheme’s ability to discriminate between binding pocket residues and non-pocket residues is comparable to Rate4Site scores which use slower Bayesian estimation for scoring residues (SDI AUC—Rate4Site:0.67, WCS:0.66; DDI AUC—Rate4Site:0.57, WCS:0.56) (Figure 2D).

#### 3.1.2. Eigenvector Centrality Score Benchmarking

We benchmarked the discriminatory power of the per-residue eigenvector centrality-based scores to the WCS and ConSurf Rate4Site scores. The eigenvector centrality-based scores show better discrimination between the pocket and non-pocket regions at the residue level compared to the WCS and ConSurf Rate4Site scores (Figure 2D). Centrality scores reveal conserved patches rather than single residues; therefore, we benchmarked their ability to pinpoint the functional regions on the SLiM-domain interactors and domain-domain interfaces in our PDB benchmarking dataset. The eigenvector centrality-based approach was benchmarked by quantifying the proportion of chains where the validated pocket overlaps with the predicted pocket. We observed that eigenvector centrality correctly identified ~70% of the validated pockets in the SDI and DDI PDB benchmarking datasets (Figure 2E). As with previous benchmarks, the active sites performed significantly better than interface datasets with 84% of the expected pockets rediscovered. As many structural modules will have multiple conserved interaction surfaces, we benchmarked the centrality approach over multiple iterations showing, as expected, increasing recall with each iteration (Figure 2F and Figure S2). Next, we compared the conservation of the validated pocket in the PDB benchmarking datasets to the quality of the return xProtCAS conserved accessible surfaces. We observed that when the known interface surface is highly conserved relative to the rest of the structural unit surface, xProtCAS is more likely to pinpoint the correct surface and the overlap with the known surface interface is higher (Figure 2G). Finally, we compared the conserved accessible surfaces returned by the xProtCAS and PatchFinder [15,16] from the PDB benchmarking datasets. The surfaces extracted by xProtCAS were slightly smaller in size than PatchFinder surfaces, but when compared to known pockets we observed that xProtCAS had slightly better precision and recall for both the SDI (PatchFinder—precision: 0.34, recall:0.29; xProtCAS—precision: 0.41, recall:0.32) and DDI (PatchFinder—precision: 0.21, recall:0.13; xProtCAS—precision: 0.41, recall:0.18) datasets (Table S4). Most importantly, there is a significant difference in the running time of the two tools (Table S4) favouring the centrality-based approach.

### 3.2. Potential Novel Interfaces in the Human Proteome

**Figure 3:**
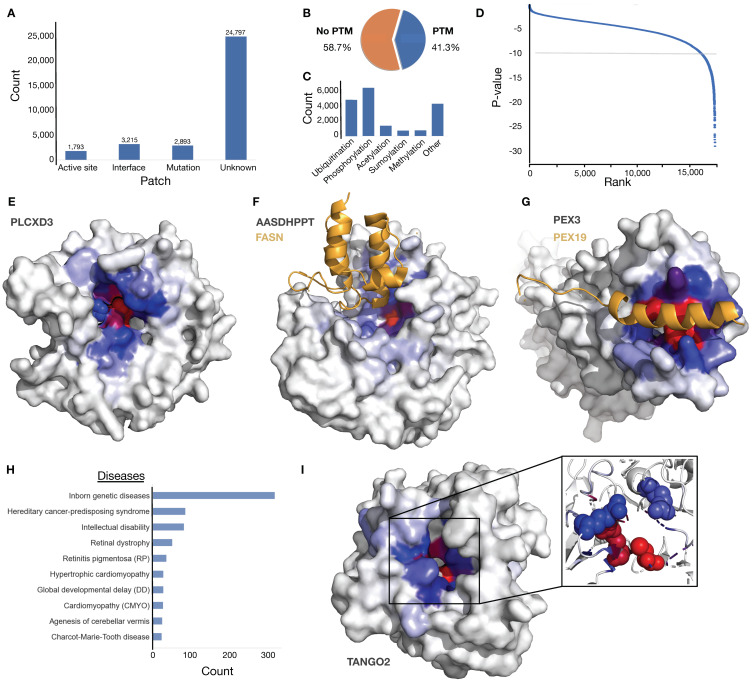
Descriptive information on the human proteome analysis. (**A**) A bar plot showing the overlap of the most conserved accessible surfaces from the 31,702 structural modules of the human proteome with 3 intersecting groups (active sites, known interfaces, regions with clinical mutation/s) or a fourth separate group of the conserved accessible surfaces without any functional annotation. (**B**) A pie chart showing the distribution of the conserved accessible surfaces with and without overlapping PTMs. (**C**) A bar plot showing the most frequent modifications overlapping with the 31,702 conserved accessible surfaces. (**D**) Ranked scatterplot of the *relative patch conservation p-value* for the 17,477 PAE-filtered conserved accessible surfaces. The *1.0 × 10^−10^* cut-off is denoted by a light grey line. (**E**) PI-PLC X domain-containing protein 3 (PLCXD3, Q63HM9) coloured by eigenvector centrality scores (colour scheme starts with grey for low scores, blue for average scores, and red for high scores, the same colour scheme is used in all remaining structure panels in the figure) with active sites on residues 37H and 114H overlapping the most conserved accessible surface (*p*-value: 1.46 × 10^−11^). (**F**) Structure of AlphaFold2 model of L-aminoadipate-semialdehyde dehydrogenase-phosphopantetheinyl transferase (AASDHPPT, Q9NRN7, grey) coloured by eigenvector centrality scores showing the overlap of the most conserved accessible surface (*p*-value: 4.62 × 10^−10^) with a domain-domain interface with fatty acid synthase (FASN, P49327, orange) (PDB ID: 2CG5). (**G**) Structure of Peroxisomal biogenesis factor 3 (PEX3, P56589, grey) with a known SLiM-domain interface with Peroxisomal biogenesis factor 19 (PEX19, P40855, orange) overlapping the most conserved accessible surface (*p*-value: 9.14 × 10^−10^) (PDB ID: 3AJB). (**H**) The most frequent diseases related to mutations overlapping the 31,702 conserved accessible surfaces. (**I**) Structure of AlphaFold2 model of Transport and Golgi organisation protein 2 homolog (TANGO2, Q6ICL3, grey) coloured by eigenvector centrality scores highlighting four clinically significant mutations at residues 2, 26, 32, and 88 (sphere representations in inlay) in the core of the conserved accessible surface (*p*-value: 1.30 × 10^−10^).

The xProtCAS pipeline was applied to 20,395 UniProt-reviewed human proteins to define a set of potential novel interfaces in the human proteome. The autonomous structural unit definition resulted in 31,702 autonomous structural modules (Table S5). The top-ranked conserved accessible surface on each subunit was extracted and annotated for overlap with relevant functional information. Of the 31,702 conserved accessible surfaces, 1793 (5.6%) are identified with active sites, 3215 (10.1%) intersect known interfaces and 2893 (9.1%) with clinically significant mutations of which only 820 are known active sites or interfaces (Figure 3A). The majority of surfaces (24,797, 78.2%) had no overlapping annotation. We also observed a large number of the surfaces overlapped with post-translational modification, with phosphorylation and ubiquitination representing the most common modifications (Figure 3B,C).

Next, we filtered the set for high-accuracy structural modules using the mean Predicted Aligned Error (PAE), leaving approximately half of the structural modules (17,477 of 31,702 with PAE less than 5Å). The high-accuracy structural module set was filtered by *relative patch conservation p-value* (cut-off of *1.0 × 10^−10^*, representative examples of structural modules with varying *relative patch conservation p-value* scores are available in Supplementary Figure S3) to define the set of conserved accessible surfaces with the most significant difference between the pocket and non-pocket surface residues (Figure 3D). The remaining 1406 structural modules showed significant enrichment of functional annotation compared to the complete dataset with 406 (28.8%) active sites, 275 (19.6%) known interfaces, and 320 (22.8%) clinically significant mutations, however, 621 (44.2%) structural modules still had no overlapping annotation and represent highly conserved and uncharacterised conserved accessible surfaces.

Figure 3E–G,I provide a set of representative examples of both characterised and uncharacterised conserved accessible surfaces. The benchmarking results showed that xProtCAS performed strongly at mapping surfaces overlapping active sites of enzymes. The returned surface in PI-PLC X domain-containing protein 3 (PLCXD3) overlaps the active sites residues 37H and 114H with phosphoric diester hydrolase activity (Figure 3E) [41,58]. The xProtCAS tool can also pinpoint functional surfaces that drive protein-protein interactions. The returned surface on L-aminoadipate-semialdehyde dehydrogenase-phosphopantetheinyl transferase (AASDHPPT) represents a known domain-domain interface with the fatty acid synthase (FASN) (Figure 3F) [59]. Similarly, a highly conserved surface on Peroxisomal biogenesis factor 3 (PEX3) represents a SLiM binding pocket which contributes to the assembly of membrane vesicles and by acting as a docking surface for Peroxisomal biogenesis factor 19 (PEX19) (Figure 3G) [60]. Many of the returned surfaces overlapped clinically significant mutations linked to a wide variety of diseases (Figure 3H). For example, the most conserved surface on Transport and Golgi organisation protein 2 homolog (TANGO2) has four clinically significant mutations linked to *metabolic crises, recurrent, with rhabdomyolysis, cardiac arrhythmias, and neurodegeneration* (MECRCN), and it is not characterised as a known interface or active site (Figure 3I).

## 4. The xProtCAS Web Server

**Figure 4:**
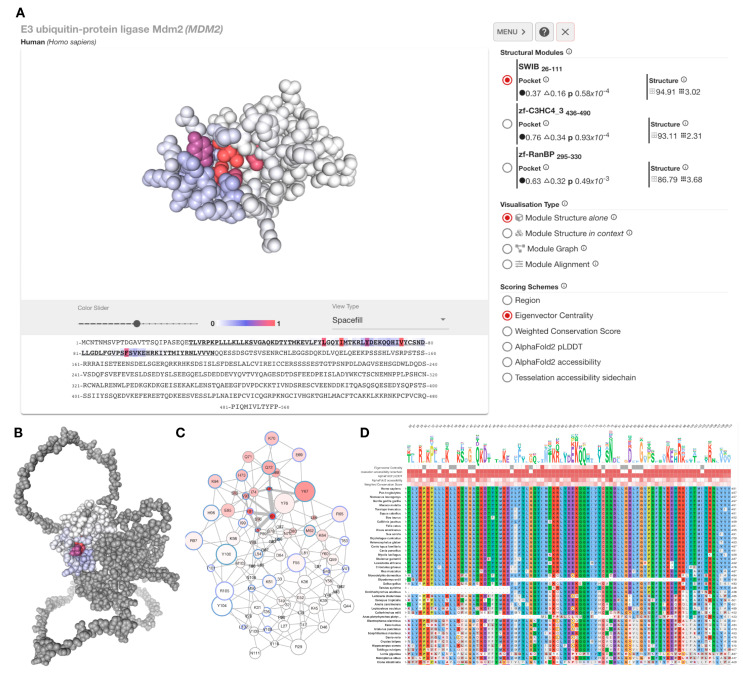
Overview of xProtCAS web server. (**A**) The results analysis page of MDM2 consists of an interactive 3D viewer, a sequence viewer and a side menu containing the structural modules of the protein, the conserved accessible surface metrics for the structural modules, and a set of visualisation options. (**B**) The SWIB structural module of MDM2 in the context of the full-length protein coloured by weighted conservation scores (WCS). (**C**) A graph representation of the SWIB domain structural module of MDM2. The graph displays each residue as a node represented with a circle in the graph; the filling colour of the circle reflects the chosen scoring scheme, and the blue colour of the circles’ circumference shows if the residue is a contacting residue in any known interface. The circle size reflects the residue’s accessibility. Neighbouring nodes are connected with edges where edge thickness represents centrality scores. (**D**) Multiple sequence alignment view of the SWIB domain structural module of MDM2. A variant of the Clustal X Colour Scheme is used to colour residues. The default scheme colours are used, in addition, negative BLOSUM64 scoring positions are coloured grey and charged residue changes are denoted by circles. The sequence logo shows the composition of the alignment column of each position scaled by residue accessibility to emphasise more conserved and accessible residues. Each position is hoverable and reveals a tooltip with a detailed description.

The xProtCAS pipeline and interactive visualisations have been made available as a web server at http://slim.icr.ac.uk/projects/xprotcas. Proteins can be searched using protein name, gene name, or UniProt accession. The analysis page of the query protein (Figure 4A) provides an interactive viewer to display the defined structural units and conserved accessible surfaces. The sidebar provides a list of structural modules and metrics related to their conserved accessible surface. The server can display a selected structural module separately or in the context of the full-length protein (Figure 4B) with structure (Figure 4A), graph (Figure 4C), and multiple sequence alignment (Figure 4D) representations. The structure and structure representations allow various colouring schemes to colour residues based on centrality, conservation, or accessibility scores. The multiple sequence alignments used in the conservation score calculation are shown in the Module Alignments section of the web interface. All data can be downloaded in the downloads section in JavaScript Object Notation (JSON) format.

## 5. Conclusions

Residue conservation can indicate a function that has been maintained across divergent sequences. Consequently, the variability of conservation across a primary sequence or surface can be leveraged to identify functionally important residues. In this work, we have designed an approach for conserved protein surface annotation that encodes the accessibility, topology, and conservation of a protein as a graph. The nodes of the graph are accessible residues in a structural module, the three-dimensional topology of the structural module is encoded in the edges of the graph, and edge weights are used to encode residue conservation scores of the connected residue nodes. Eigenvector centrality gives higher scores to nodes with influential neighbours; as a result, subgraphs with high eigenvector centrality scoring residues reflect surfaces with a high concentration of relatively strongly conserved residues. We have shown that by applying eigenvector centrality to integrate the topological, accessibility, and evolutionary information encoded in the graph we can pinpoint conserved accessible surfaces, and these surfaces strongly correlate with functional surfaces on a protein. We introduced evaluation scores to rank and quantify confidence in a given surface, and we demonstrated these scores are strong discriminators for conserved accessible surfaces that overlap a known interface. In the future, integrating data from non-conservation approaches for pocket discovery with the conservation-based eigenvector centrality approach, for example, using machine learning, could significantly improve the quality of the predictions of either approach alone.

The rapid advances in deep learning methods for protein structure prediction have resulted in an explosion of high-quality structural models of proteins. In this study, we take advantage of AlphaFold2 structural models to perform evolutionary analyses on a huge set of proteins previously inaccessible for structure-conservation exploration. The direct integration of AlphaFold2 structural models into the pipeline simplifies access to the complete protein search space. The speed of the pipeline, in the range of seconds when the structure and alignment are locally available, boosts its scalability and allows proteome-wide analysis to be performed with ease. As a result, we have explored the evolutionary landscape of human protein surfaces, finding thousands of putative binding pockets without a known function in need of further experimental exploration. However, caution should be taken for surface evolution analyses of AlphaFold2 data. Models have variable levels of quality, and some modules may have partial local misfolding or buried residue side chains incorrectly appearing on the protein surface. Given the higher level of conservation of these residues, it is important to be aware of the AlphaFold2 confidence metrics and consider them when analysing returned surfaces. We utilise two scores to quantify the structure quality of the patch represented in the mean patch predicted Local Distance Difference Test (pLDDT) and Predicted Aligned Error (PAE). Both scores are AlphaFold metrics for scoring structure prediction confidence and accuracy.

The xProtCAS web server represents a fast, simple, and intuitive tool to analyse protein surface conservation. The two comparable available web-based tools for conserved accessible surface discovery, PatchFinder, and FuncPatch web servers, were no longer functional at the time of publication. There are overlaps with the functionality of the ConSurf server. However, the definition of the most conserved accessible surface and integration with AlphaFold2 models of the xProtCAS server adds key functionality not available with the ConSurf server. The xProtCAS server uses AlphaFold2 models as input. In our experience, the full-length AlphaFold2 structures reduce noise resulting from intramolecular interaction surfaces that are uncomplexed when domains are characterised independently. Furthermore, when an experimental structure is available and has been used in training, the AlphaFold2 model rarely diverges significantly from the experimental structure. However, if a non-AlphaFold2-derived structure is required the standalone software is freely available. An additional use case of the standalone software is to define multiple surfaces. Structural modules with multiple functional surfaces are an issue for many reasons. Firstly, the xProtCAS pipeline can find functional protein interaction surfaces yet incur a penalty in benchmarking if the surface is not part of the testing set. This also makes the definition of a negative set difficult. Secondly, as the centrality-based approach returns the most conserved surface in the graph, highly conserved surfaces can be discarded. The xProtCAS pipeline can be applied in an iterative manner to remedy this issue by removing the most conserved surface with each iteration. The number of iterations can be constrained by applying a *relative patch conservation p-value* cut-off to return significantly conserved accessible surfaces.

In summary, we have developed xProtCAS, a graph-based pipeline to define conserved accessible surfaces in protein structures. The xProtCAS pipeline provides a novel tool to the biological community that allows rapid analysis of the surface properties of a protein to define putative functional pockets, pinpoint potential interaction interfaces, aid in experimental design, and prioritise proteins for functional characterisation.

## Data Availability

Data generated in this analysis is available in the Supplementary Materials.

## Supplementary Materials

The following supporting information can be downloaded at: https://www.mdpi.com/article/10.3390/biom13060906/s1, Table S1: List of human E3 ligases manually collected from the literature and used in tuning the resolution parameter of the community detection algorithm; Table S2: Benchmarking of accessible residues settings; Table S3: PDB dataset used in evaluating xProtCAS’s ability to find functional regions. The dataset consists of SLiM-domain interactors (SDI), domain-domain interactors (DDI), and their active sites; Table S4: Comparison between functional patches extracted by 2 different approaches, PatchFinder and xProtCAS. The 2 approaches are tested to find residues in the SLiM-domain and domain-domain interfaces; Table S5: Dataset of 31,702 structural subunits of human proteins sorted with the *p*-values of the top-ranked patches. The dataset represents the results of running xProtCAS on 20,395 proteins of the Human Proteome. References [61,62] are cited in the Supplementary Materials. Figure S1. The number of proteins with Pfam domains distributed amongst multiple predicted communities when using resolutions starting from 1 and going down to 0 with 0.01 as a step at a time. We chose 0.4 as the default resolution at which 90% of proteins are large enough not to have Pfam domains distributed amongst multiple communities; Figure S2. Iterations of xProtCAS on five examples of E3 ligases until it finds the correct degron-binding pocket validated manually from literature and automatically using complex structures based on the closeness between heavy atoms (when the degron chain is present in the PDB structure file). Residues with white colour have low centrality scores, yellow colour for average scores, red for high scores, and yellow represents the degron residues of the interacting partner; Figure S3. (A) Procollagen galactosyltransferase 1 (Q8NBJ5) structure displaying conservation scores on the top figure (grey indicates low score, blue average score, and red high scores) and a highly significant top-ranked predicted patch on the bottom figure (the red color represent the defined patch, the grey color for the rest of the surface) (*p*-value: 1.29 × 10^−18^) making it ranks at the head of the list of all patches on human proteins (rank: 343). (B) Alanine–glyoxylate aminotransferase 2 mitochondrial (Q9BYV1) structure with the top-ranked patch (on the bottom figure) having average ranking (rank: 14,875) in the list of patches on all human proteins, as the region surrounding the predicted patch is also conserved (on the top figure) which affected the patch conservation significance (*p*-value: 9.01 × 10^−6^). (C) SLIT and NTRK-like protein 5 (O94991) structure with an equally conserved surface (on the top figure) leading to the top-ranked patch being barely significant (*p*-value: 0.002) and ranks in the tail of the list of all human protein patches (rank: 27, 898).
