# Supplementary material for: xProtCAS: A Toolkit for Extracting Conserved Accessible Surfaces from Protein Structures
Source: Biomolecules. 2023 May 30;13(6):906. doi: 10.3390/biom13060906 (PMC10296640; doi:10.3390/biom13060906)
Supplement: Supplementary file 1 [file biomolecules-13-00906-s001.zip › Supplementary Material.pdf]

Supplementary Notes

Supplementary note 1. Processing of PDB to extract non-redundant SLiM-containing structures

We have collected a set of 522 non-redundant pairwise SLiM-domain interfaces in resolved human complex structures.

Structures were processed as follows:

- Download and screen all PDB structures that contain a biological assembly with 2+ chains.
- Define disordered peptides in structures of protein complexes using FLIPPER [61].
- Extract SLiMs using the discriminator of bound footprint <1500 $Å^2$, mono-partite interface as defined by a single distinct secondary structural properties in the bound conformation, and defined by FLIPPER as disordered across the entire chain.

SLiM containing PDB structures were processed by CD-HIT [62], sequences were split at 30% sequence identity, and one representative member was selected from each cluster to remove redundancy.

Supplementary note 2. Databases with different levels of evolutionary divergence

Quest For Orthologs (QfO): Anopheles gambiae, Aquifex aeolicus (strain VF5), Arabidopsis thaliana, Bacillus subtilis (strain 168), Bacteroides thetaiotaomicron (strain ATCC 29148 / DSM 2079 / JCM 5827 / CCUG 10774 / NCTC 10582 / VPI-5482 / E50), Bos taurus, Bradyrhizobium diazoefficiens (strain JCM 10833 / BCRC 13528 / IAM 13628 / NBRC 14792 / USDA 110), Branchiostoma floridae, Caenorhabditis elegans, Candida albicans (strain SC5314 / ATCC MYA-2876), Canis lupus familiaris, Chlamydia trachomatis (strain D/UW-3/Cx), Chloroflexus aurantiacus (strain ATCC 29366 / DSM 635 / J-10-fl), Ciona intestinalis, Cryptococcus neoformans var. neoformans serotype D (strain JEC21 / ATCC MYA-565), Danio rerio, Deinococcus radiodurans (strain ATCC 13939 / DSM 20539 / JCM 16871 / LMG 4051 / NBRC 15346 / NCIMB 9279 / R1 / VKM B-1422), Dictyoglomus turgidum (strain DSM 6724 / Z-1310), Dictyostelium discoideum, Drosophila melanogaster, Escherichia coli (strain K12), Fusobacterium nucleatum subsp. nucleatum (strain ATCC 25586 / DSM 15643 / BCRC 10681 / CIP 101130 / JCM 8532 / KCTC 2640 / LMG 13131 / VPI 4355), Gallus gallus, Geobacter sulfurreducens (strain ATCC 51573 / DSM 12127 / PCA), Giardia intestinalis (strain ATCC 50803 / WB clone C6), Gloeobacter violaceus (strain ATCC 29082 / PCC 7421), Halobacterium salinarum (strain ATCC 700922 / JCM 11081 / NRC-1), Homo sapiens, Ixodes scapularis, Korarchaeum cryptofilum (strain OPF8), Leishmania major, Leptospira interrogans serogroup Icterohaemorrhagiae serovar Lai (strain 56601), Macaca mulatta, Methanocaldococcus jannaschii (strain ATCC 43067 / DSM 2661 / JAL-1 / JCM 10045 / NBRC 100440), Methanosarcina acetivorans (strain ATCC 35395 / DSM 2834 / JCM 12185 / C2A), Monodelphis domestica, Monosiga brevicollis, Mus musculus, Mycobacterium tuberculosis (strain ATCC 25618 / H37Rv), Nematostella vectensis, Aspergillus fumigatus (strain ATCC MYA-4609 / CBS 101355 / FGSC A1100 / Af293), Neurospora crassa (strain ATCC 24698 / 74-OR23-1A / CBS 708.71 / DSM 1257 / FGSC 987), Ornithorhynchus anatinus, Pan troglodytes, Phaeosphaeria nodorum (strain SN15 / ATCC MYA-4574 / FGSC 10173), Physcomitrium patens, Plasmodium falciparum (isolate 3D7), Pseudomonas aeruginosa (strain ATCC 15692 / DSM 22644 / CIP 104116 / JCM 14847 / LMG 12228 / 1C / PRS 101 / PAO1), Rattus norvegicus, Rhodopirellula baltica (strain DSM 10527 / NCIMB 13988 / SH1), Saccharomyces cerevisiae (strain ATCC 204508 / S288c), Schistosoma mansoni, Schizosaccharomyces pombe (strain 972 / ATCC 24843), Sclerotinia sclerotiorum (strain ATCC 18683 / 1980 / Ss-1), Streptomyces coelicolor (strain ATCC BAA-471 / A3(2) / M145), Saccharolobus solfataricus (strain ATCC 35092 / DSM 1617 / JCM 11322 / P2), Synechocystis sp. (strain PCC 6803 / Kazusa), Takifugu rubripes, Thalassiosira pseudonana, Thermococcus kodakarensis (strain ATCC BAA-918 / JCM 12380 / KOD1), Thermodesulfovibrio yellowstonii (strain ATCC 51303 / DSM 11347 / YP87), Thermotoga

maritima (strain ATCC 43589 / DSM 3109 / JCM 10099 / NBRC 100826 / MSB8), Trichomonas vaginalis, Ustilago maydis (strain 521 / FGSC 9021), Xenopus tropicalis, Yarrowia lipolytica (strain CLIB 122 / E 150).

Metazoa: Acyrthosiphon pisum, Adineta vaga, Aedes aegypti, Amphimedon queenslandica, Anopheles darlingi, Anopheles gambiae, Anoplophora glabripennis, Apis mellifera, Atta cephalotes, Belgica antarctica, Bombus impatiens, Bombus terrestris, Bombyx mori, Branchiostoma lanceolatum, Brugia malayi, Caenorhabditis elegans, Capitella teleta, Crassostrea gigas, Culex quinquefasciatus, Danaus plexippus, Daphnia pulex, Dendroctonus ponderosae, Dinothrombium tinctorium, Drosophila melanogaster, Folsomia candida, Heliconius melpomene, Helobdella robusta, Hofstenia miamia, Ixodes scapularis, Leptotrombidium deliense, Lingula unguis, Loa loa, Lottia gigantea, Lucilia cuprina, Mayetiola destructor, Megaselia scalaris, Melitaea cinxia, Mnemiopsis leidyi, Nasonia vitripennis, Nematostella vectensis, Octopus bimaculoides, Onchocerca volvulus, Orchesella cincta, Pediculus humanus subsp. corporis, Rhodnius prolixus, Sarcoptes scabiei, Schistosoma mansoni, Solenopsis invicta, Stegodyphus mimosarum, Strigamia maritima, Strongylocentrotus purpuratus, Strongyloides ratti, Teleopsis dalmanni, Tetranychus urticae, Thelohanellus kitauei, Tribolium castaneum, Trichinella spiralis, Trichoplax adhaerens, Zootermopsis nevadensis, Ciona intestinalis, Ornithorhynchus anatinus, Phascolarctos cinereus, Monodelphis domestica, Equus caballus, Myotis lucifugus, Sus scrofa, Bos taurus, Tursiops truncatus, Panthera pardus, Felis catus, Ursus americanus, Canis lupus familiaris, Otolemur garnettii, Prolemur simus, Carlito syrichta, Callithrix jacchus, Nomascus leucogenys, Pan troglodytes, Homo sapiens, Gorilla gorilla gorilla, Macaca mulatta, Marmota marmota marmota, Mus musculus, Cricetulus griseus, Cavia porcellus, Heterocephalus glaber, Dipodomys ordii, Oryctolagus cuniculus, Loxodonta africana, Anolis carolinensis, Sphenodon punctatus, Latimeria chalumnae, Chrysemys picta bellii, Dromaius novaehollandiae, Serinus canaria, Gallus gallus, Anas platyrhynchos platyrhynchos, Crocodylus porosus, Xenopus tropicalis, Esox lucius, Ictalurus punctatus, Electrophorus electricus, Danio rerio, Hippocampus comes, Oryzias latipes, Takifugu rubripes, Cottoperca gobio, Scophthalmus maximus, Monopterus albus, Clupea harengus, Lepisosteus oculatus, Eptatretus burgeri, Callorhinchus milii.

Vertebrata: Ciona intestinalis, Ornithorhynchus anatinus, Phascolarctos cinereus, Monodelphis domestica, Equus caballus, Myotis lucifugus, Sus scrofa, Bos taurus, Tursiops truncatus, Panthera pardus, Felis catus, Ursus americanus, Canis lupus familiaris, Otolemur garnettii, Prolemur simus, Carlito syrichta, Callithrix jacchus, Nomascus leucogenys, Pan troglodytes, Homo sapiens, Gorilla gorilla gorilla, Macaca mulatta, Marmota marmota marmota, Mus musculus, Cricetulus griseus, Cavia porcellus, Heterocephalus glaber, Dipodomys ordii, Oryctolagus cuniculus, Loxodonta africana, Anolis carolinensis, Sphenodon punctatus, Latimeria chalumnae, Chrysemys picta bellii, Dromaius novaehollandiae, Serinus canaria, Gallus gallus, Anas platyrhynchos platyrhynchos, Crocodylus porosus, Xenopus tropicalis, Esox lucius, Ictalurus punctatus, Electrophorus electricus, Danio rerio, Hippocampus comes, Oryzias latipes, Takifugu rubripes, Cottoperca gobio, Scophthalmus maximus, Monopterus albus, Clupea harengus, Lepisosteus oculatus, Eptatretus burgeri, Callorhinchus milii.

Mammalia: Loxodonta africana, Trichechus manatus latirostris, Oryctolagus cuniculus, Dipodomys ordii, Heterocephalus glaber, Cavia porcellus, Cricetulus griseus, Mus musculus, Rattus norvegicus, Ictidomys tridecemlineatus, Cercocebus atys, Macaca mulatta, Gorilla gorilla gorilla, Homo sapiens, Pan troglodytes, Pongo abelii, Nomascus leucogenys, Aotus nancymaae, Callithrix jacchus, Carlito syrichta, Canis lupus familiaris, Odobenus rosmarus divergens, Leptonychotes weddellii, Ailuropoda melanoleuca, Ursus americanus, Felis catus, Balaenoptera acutorostrata scammoni, Tursiops truncatus, Bos taurus, Ovis aries, Sus scrofa, Myotis lucifugus, Erinaceus europaeus, Equus caballus, Sarcophilus harrisii, Monodelphis domestica, Ornithorhynchus anatinus, Ciona intestinalis, Ciona savignyi.

Supplementary Figures

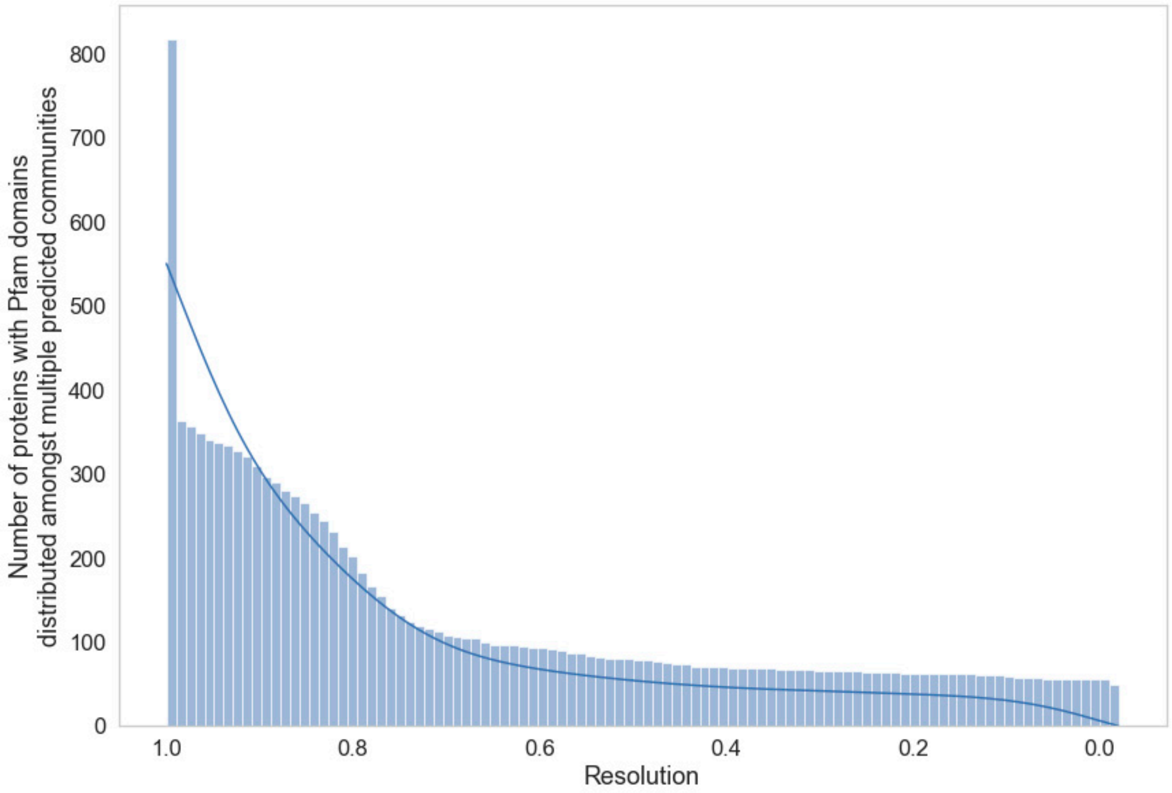

Figure S1. The number of proteins with Pfam domains distributed amongst multiple predicted communities when using resolutions starting from 1 and going down to 0 with 0.01 as a step at a time. We chose 0.4 as the default resolution at which ~90% of proteins are large enough not to have Pfam domains distributed amongst multiple communities.

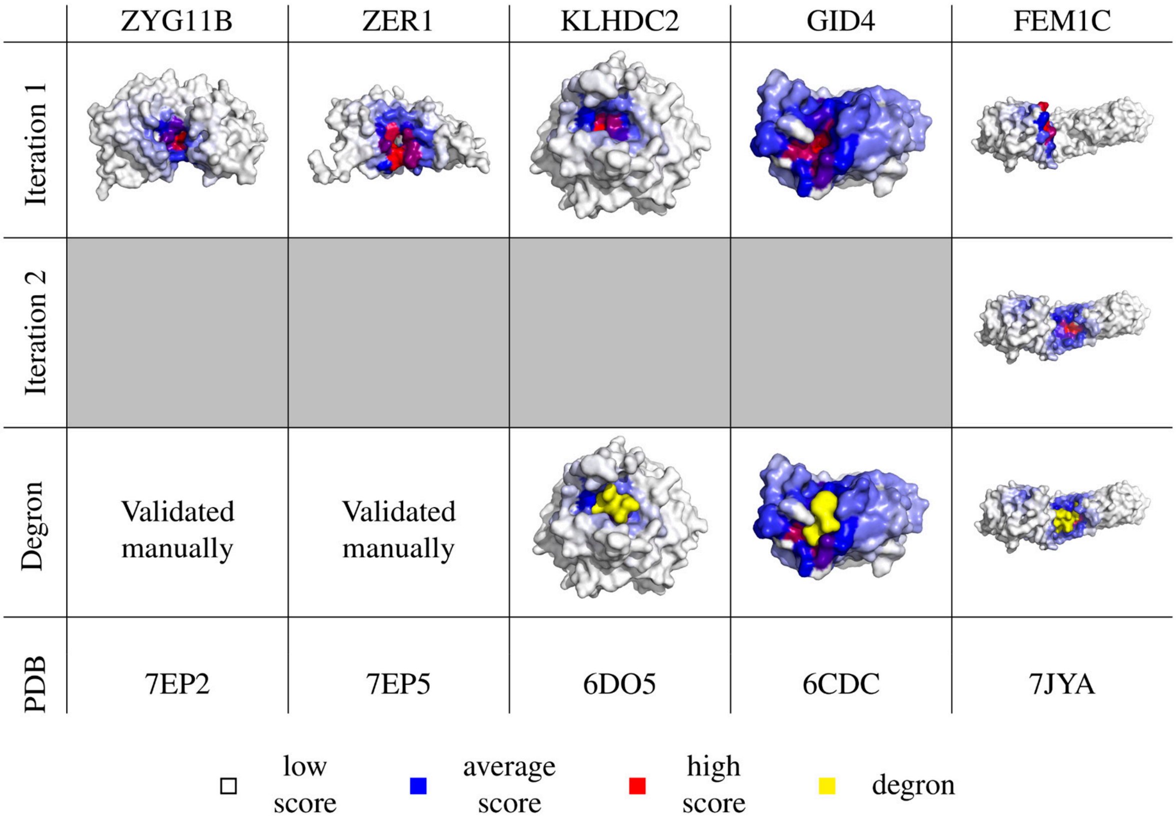

Figure S2. Iterations of xProtCAS on five examples of E3 ligases until it finds the correct degron-binding pocket validated manually from literature and automatically using complex structures based on the closeness between heavy atoms (when the degron chain is present in the PDB structure file). Residues with white colour have low centrality scores, yellow colour for average scores, red for high scores, and yellow represents the degron residues of the interacting partner.

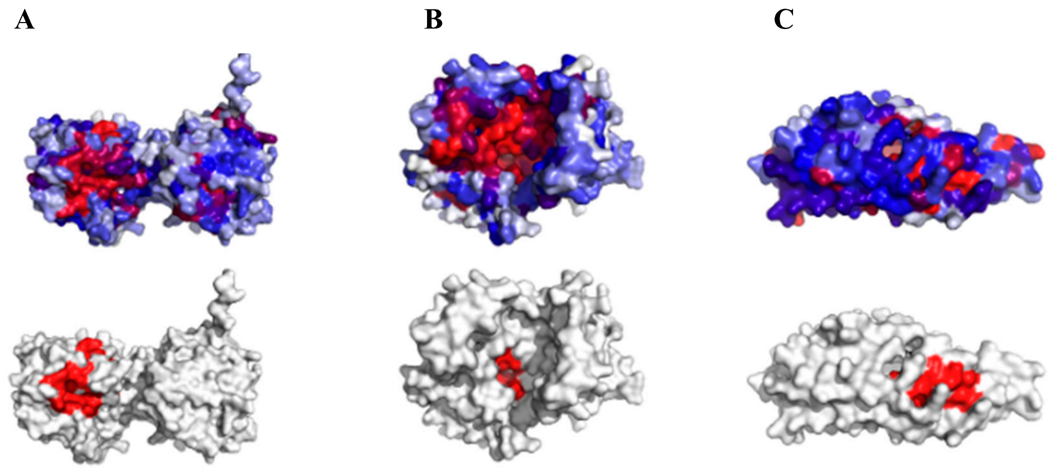

Figure S3. A) Procollagen galactosyltransferase 1 (Q8NBJ5) structure displaying conservation scores on the top figure (grey indicates low score, blue average score, and red high scores) and a highly significant top-ranked predicted patch on the bottom figure (the red color represent the defined patch, the grey color for the rest of the surface)(p-value: 1.29×10^{-18}) making it ranks at the head of the list of all patches on human proteins (rank: 343). B) Alanine--glyoxylate aminotransferase 2 mitochondrial (Q9BYV1) structure with the top-ranked patch (on the bottom figure) having average ranking (rank: 14,875) in the list of patches on all human proteins, as the region surrounding the predicted patch is also conserved (on the top figure) which affected the patch conservation significance (p-value: 9.01×10^{-6}). C) SLIT and NTRK-like protein 5 (O94991) structure with an equally conserved surface (on the top figure) leading to the top-ranked patch being barely significant (p-value: 0.002) and ranks in the tail of the list of all human protein patches (rank: 27, 898).
