# Supplementary material for: xProtCAS: A Toolkit for Extracting Conserved Accessible Surfaces from Protein Structures
Source: Biomolecules. 2023 May 30;13(6):906. doi: 10.3390/biom13060906 (PMC10296640; doi:10.3390/biom13060906)
Supplement: Supplementary file 1 [file biomolecules-13-00906-s001.zip › Table_S2.pdf]

Table S2. Benchmarking of different settings to define accessible residues. Columns in order: Atom type determines whether accessibility definition is based on any atom or only side chain atoms; The number of atoms is the least number of accessible atoms to consider the residue as accessible; The mean size of predicted patch; The mean size of actual interface; The mean intersections between the patch and interface; The mean total number of accessible residues per chain; The mean of recall (intersection/interface); The mean of precision (intersection/patch); The mean ratio between patch size and all accessible residues; Percentage of chains in the dataset with a non-zero intersection.

Atoms type	# atoms	Predicted patch	Actual interface	Intersection	# Residues	Recall	Precision	to all	Percentage
SLiM-domain interfaces									
side	1	21	23	7	209	0.33	0.42	0.14	70
side	2	17	19	6	166	0.32	0.42	0.14	70
side	3	9	14	4	118	0.27	0.41	0.11	64
side	4	5	9	2	74	0.23	0.42	0.10	59
side	5	3	5	1	42	0.25	0.41	0.11	53
any	1	24	24	8	231	0.33	0.39	0.14	69
any	2	21	23	7	209	0.33	0.40	0.14	70
any	3	18	19	6	177	0.33	0.40	0.14	69
any	4	11	11	4	136	0.26	0.40	0.11	64
any	5	6	15	2	93	0.24	0.39	0.11	59
Domain-domain interfaces									
side	1	16	36	6	143	0.19	0.42	0.13	70
side	2	12	32	5	118	0.18	0.44	0.12	69
side	3	8	24	4	86	0.16	0.46	0.11	64
side	4	5	16	2	56	0.14	0.45	0.10	58
side	5	3	9	1	32	0.15	0.46	0.11	58
any	1	18	39	7	159	0.18	0.40	0.13	70
any	2	17	37	7	146	0.20	0.43	0.13	71
any	3	13	33	6	125	0.18	0.44	0.12	68
any	4	9	27	4	99	0.16	0.46	0.11	66

any	5	6	20	3	70	0.13	0.43	0.10	57
