# Supplementary material for: xProtCAS: A Toolkit for Extracting Conserved Accessible Surfaces from Protein Structures
Source: Biomolecules. 2023 May 30;13(6):906. doi: 10.3390/biom13060906 (PMC10296640; doi:10.3390/biom13060906)
Supplement: Supplementary file 1 [file biomolecules-13-00906-s001.zip › Table_S4.pdf]

Table S4. Comparison between functional patches extracted by 2 different approaches, PatchFinder and xProtCAS. The 2 approaches are tested to find residues in the SLiM-domain and domain-domain interfaces.

	PatchFinder	xProtCAS
Method	R4S<br>↓<br>PatchFinder<br>(Heuristic algorithm)<br>↓<br>Patch	WCS<br>↓<br>Eigenvector Centrality and Hierarchical clustering<br>↓<br>Patch
Speed	Slow (Minutes)	Fast (Milliseconds)
SLiM-domain interfaces		
Patch size mean	25	21
Patch size std	32	20
Precision mean	0.34	0.41
Recall mean	0.29	0.32
Domain-domain interfaces		
Patch size mean	23	16
Patch size std	21	12
Precision mean	0.21	0.41
Recall mean	0.13	0.18
